# A pilot study: a teaching electronic medical record for educating and assessing residents in the care of patients

**DOI:** 10.1080/10872981.2018.1447211

**Published:** 2018-03-06

**Authors:** Joshua Smith, W. Graham Carlos, Cynthia S. Johnson, Blaine Takesue, Debra Litzelman

**Affiliations:** ^a^ Division of Allergy, Pulmonary and Critical Care Medicine, University of Wisconsin School of Medicine and Public Health, WI, USA; ^b^ Department of Medicine, Division of Pulmonary and Critical Care Medicine, Indiana University School of Medicine, IN, USA; ^c^ Department of Biostatistics, Indiana University, IN, USA; ^d^ Department of Medicine, Regenstrief Institute, Inc, IN, USA; ^e^ Department of Medicine, Division of General Internal Medicine, Indiana University School of Medicine, IN, USA

**Keywords:** Electronic medical record, medical education, graduate medical education, virtual patients, computer-assisted instruction, educational tool

## Abstract

**Objective**: We tested a novel, web-based teaching electronic medical record to teach and assess residents’ ability to enter appropriate admission orders for patients admitted to the intensive care unit. The primary objective was to determine if this tool could improve the learners’ ability to enter an evidence-based, comprehensive initial care plan for critically ill patients.

**Methods**: The authors created three modules using de-identifed real patient data from selected patients that were admitted to the intensive care unit. All senior residents (113 total) were invited to participate in a dedicated two-hour educational session to complete the modules. Learner performance was graded against gold standard admission order sets created by study investigators based on the latest evidence-based medicine and guidelines.

**Results**: The session was attended by 39 residents (34.5% of invitees). There was an average improvement of at least 20% in users’ scores across the three modules (Module 3-Module 1 mean difference 22.5%; *p* = 0.001 and Module 3-Module 2 mean difference 20.3%; *p* = 0.001). Diagnostic acumen improved in successive modules. Almost 90% of the residents reported the technology was an effective form of teaching and would use it autonomously if more modules were provided.

**Conclusions**: In this pilot project, using a novel educational tool, users’ patient care performance scores improved with a high level of user satisfaction. These results identify a realistic and well-received way to supplement residents’ training and assessment on core clinical care and patient management in the face of duty hour restrictions.

## Introduction

In Graduate Medical Education, new educational modalities are ever more important as trainees are allowed less time in the hospital following duty hour regulations. Medical schools have begun incorporating web-based, patient centered modules into their curriculum to ensure trainees have adequate exposure to core medical case content. These include Computer-Assisted Instruction (CAI) and virtual patients (VPs) [1,2]. CAI comes in many forms and can provide concise standardized topics to a large group of learners [1,3]. As these modalities are standardized, however, they tend to lose their realistic qualities.

VPs provide a computer program that simulates real-life clinical scenarios in which learners emulate the roles of health care providers conducting a history and physical exam and making diagnostic and therapeutic decisions [4]. VPs can be cost-intensive, with development ranging between $10,000 and $50,000, and time-intensive requiring at least 6 months to develop robust cases [2]. While the efficacy of VPs appears mixed due to the heterogeneity of study design [5], one recent study found VPs improved both baseline knowledge and long-term retention, by 25% and 15%, respectively [6]. Much of the literature regarding CAI and VPs has focused on undergraduate medical education. The role of advanced educational modalities in graduate medical education, to our knowledge, has not been well evaluated.

Electronic medical records (EMRs) have become standard of care in the USA, yet the impact of EMRs in medical education is not entirely known. Some would suggest that EMRs are deleterious to medical education, critiquing that residents and students are reporting raw data but not synthesizing or interpreting the data [7]. Given advances in healthcare, particularly implementation of EMR nationwide, it is necessary to educate students and residents in effective use of EMR as tools for patient care. Previously, institutions have created educational experiences incorporating EMR components [8,9]. The Regenstrief Institute (RI), located on the Indiana University School of Medicine campus, developed a teaching EMR (tEMR) that included real patient data that was de-identified with full CPOE. RI has extensive expertise and experience with EMRs and CPOE [10,11]. We attempted to create real-life patient experiences in which learners would be provided with a patient presentation, navigate through the teaching EMR, and ultimately enter a care plan through the computerized physician order entry (CPOE). The primary objective was determine if our tool could improve the learners’ ability to enter an evidence-based, comprehensive initial care plan for critically ill patients. Secondary objectives included determining any improvements in learner’s comfort level admitting critically ill patients and if learners found the tool to be effective.

## Methods

### Study design

**Figure 1.**
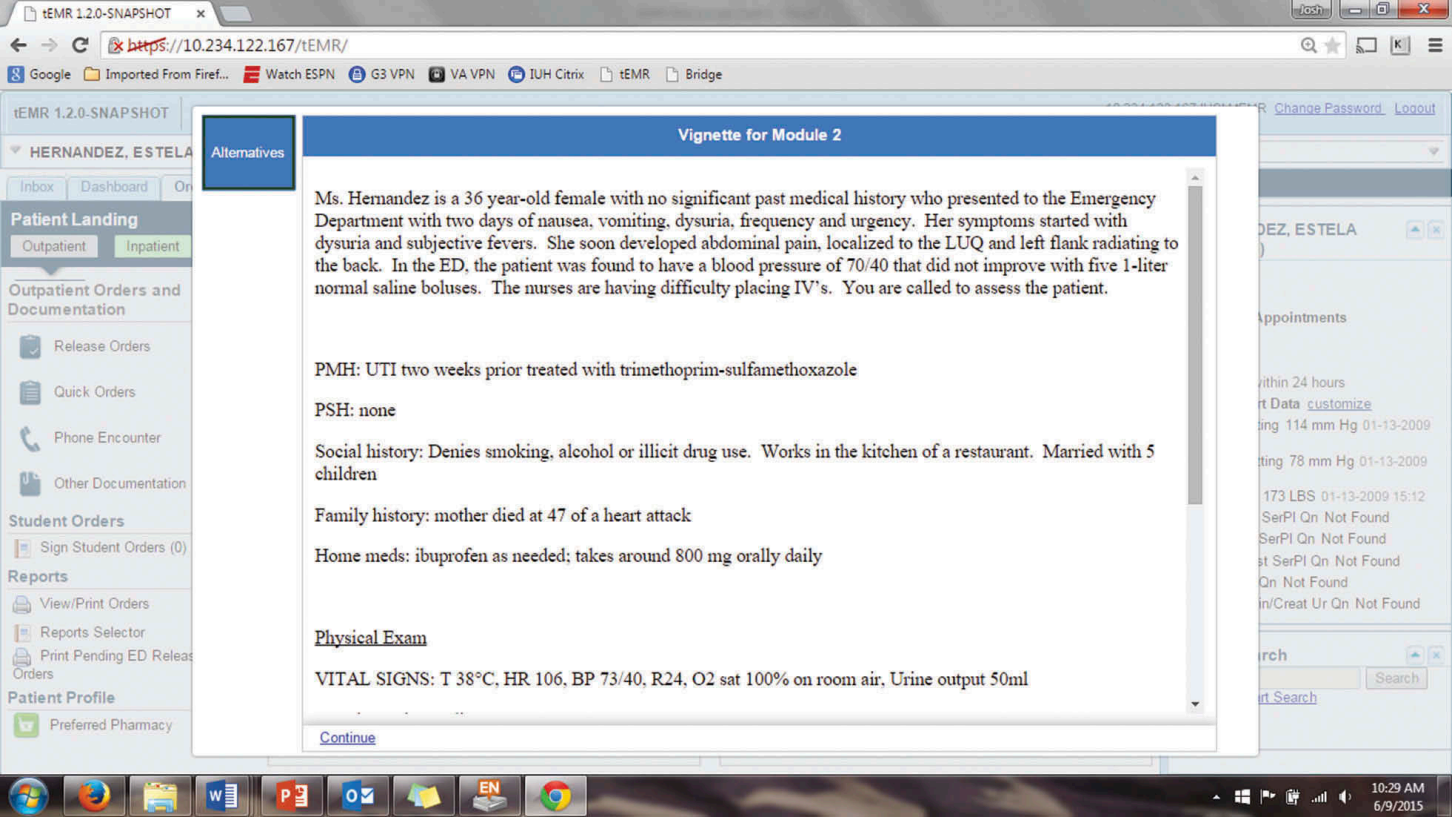
Clinical vignette provided at the module initiation.

We created three separate modules using real patients’ records that were treated in the Intensive Care Unit at our local institution with the diagnosis with severe sepsis or septic shock. The tEMR is a mirrored version of the locally developed EMR that is routinely used by all trainees at our institution. The patient records were completely de-identified and a brief clinical vignette was created highlighting patient presentation and physical exam (Figure 1). These clinical vignettes were minimally altered from the actual presentation. Available supplemental laboratory information differed between modules but was a true reflection of actual ordering practices in the Emergency Department at the time of admission. Participants were instructed to self-navigate through the tEMR and develop an initial care plan for each module. Learners received real-time feedback on their performance as well as hyperlinks to pertinent medical literature.

**Figure 2.**
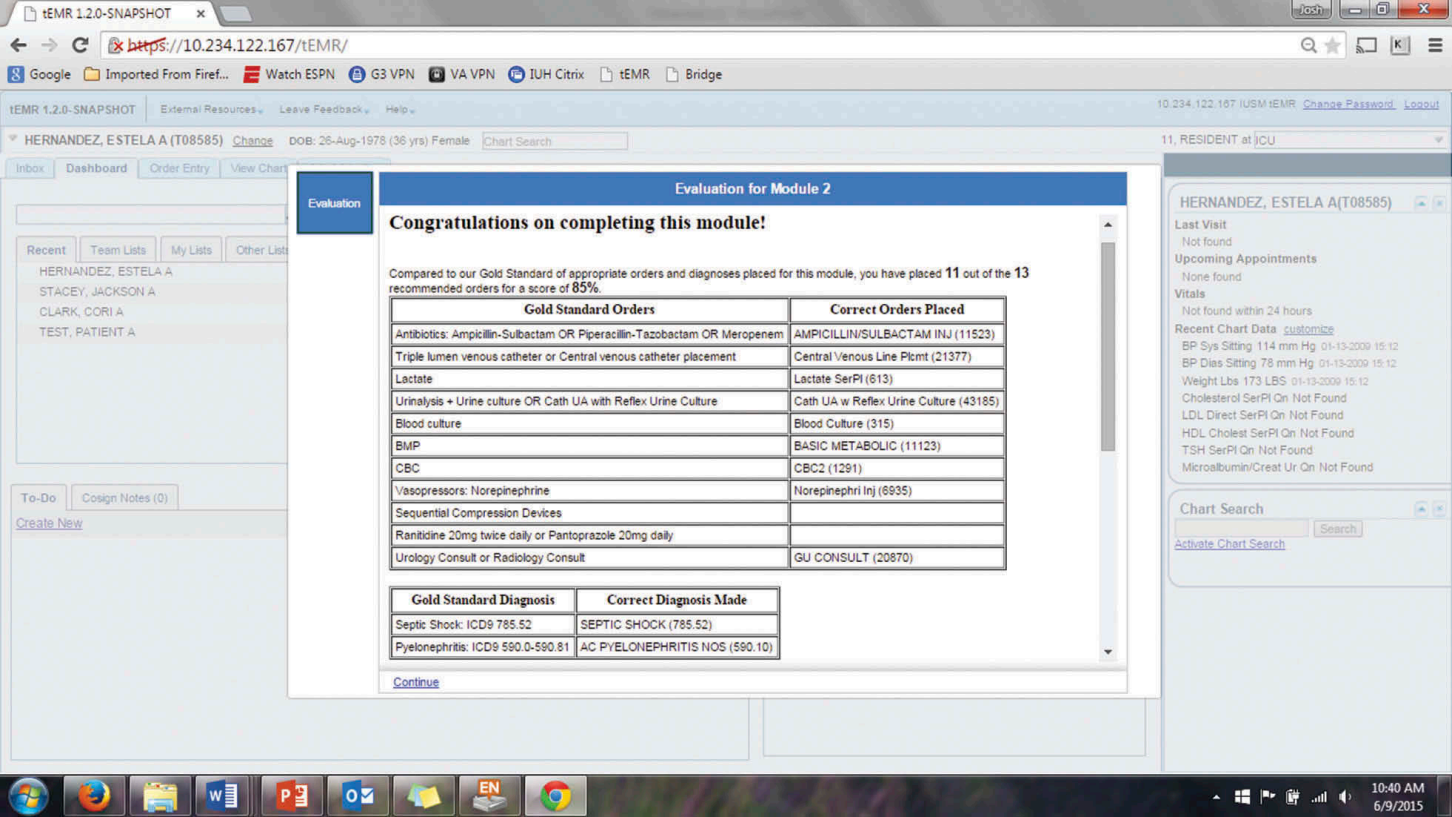
Feedback page provided at module completion.

The modules began with a clinical vignette including patient presentation and physical exam (Figure 1). The learner was expected to navigate the tEMR for pertinent past history and laboratory data. The learner then synthesized the information into a working diagnosis and created an admission order set through CPOE. Order sets were general admission orders and not focused on a specific disease. Immediately after the orders are signed into effect, they received feedback on their performance for each module compared to the gold standard (Figure 2). Learners also were provided with hyperlinks to relevant evidence-based articles for each module. At the conclusion of the two-hour session, learners were provided with a full explanation for clinical reasoning for each module. All learners progressed through modules in the same order.

### Study participants and sample size

We invited all 113 senior residents, postgraduate year (PGY) 2–4, in the Internal Medicine and Medicine-Pediatrics residencies at our institution. All senior residents received two emails from study investigators prior to the session. The modules were made available in a dedicated session in April 2015 as part of the regularly scheduled weekly educational series. The residents were provided a two-hour session in a computer lab to complete as many modules as possible. The residents were assigned a random user identifier and all information was kept anonymous and confidential to participants and researchers. At the beginning of the session, users were instructed on scoring including diagnoses and orders. This study was approved by the Internal Review Board of Indiana University-Purdue University Indianapolis

### Data collection

Learner performance was graded against gold standard admission order sets that were created by study investigators based on latest evidence-based medicine and guidelines (Appendix 1) [12,13]. Scores were calculated based on the percentage of correct orders and diagnoses placed with all orders and diagnoses required for a perfect score. There was no negative scoring for excessive or inappropriate orders. The gold standards included diagnoses, laboratory testing, procedures, medications, and necessary consultations for each patient. The complete modules including all available laboratory information, gold standard order sets, hyperlinks, and final explanation of the cases were provided to two experienced intensivists with no affiliation to the study for further validation. The validation of the modules included review of the entire module and suggested changes to the gold standard order sets and case explanation. Modules were created to be of similar complexity based on patient presentation and degree of organ failure with varying content relative to each diagnosis.

Surveys were distributed and collected immediately before and after the session. The survey tool was created and administered through REDCap (Research Electronic Data Capture), which is a secure, web-based application designed to support data capture for research studies [14]. Survey data collected included demographic information, self-reported comfort levels admitting critically ill patients, and satisfaction data related to the modules (Supplement 1).

### Data analysis

Fisher’s exact tests (FET) were used to test the association between resident year and program with module complete. Repeated measures analysis of variance was used to investigate change in scores across modules. Resident was a random effect and module pair was a fixed effect. When there was a significant change over time, pair-wise tests were done to determine which modules significantly differed. Tukey’s adjustment for multiple comparisons was used. To examine the effect of year, number of previous ICU rotations, and time since last ICU rotation, additional models were fit including each of the covariates. The repeated measures analyses were repeated including only residents with complete data. McNemar’s test was used to test change in residents’ comfort level from pre- to post- session.

## Results

Thirty-nine residents (34.5% of residents invited) attended the session with all 39 completing the first module. Thirty-two (82.1%) and 19 (48.7%) residents completed modules 2 and 3, respectively. The rate of module completion among residents was related to multiple factors including clinical obligations limiting time in the teaching lab and server connectivity issues with the tEMR. Among the residents who completed all modules, there were 8 PGY2s, 8 PGY3s, and 3 PGY4s. There were 15 Internal Medicine residents and 4 Medicine-Pediatrics residents. Module completion did not significantly differ by year or program (FET *p* = 0.65 and *p* = 0.38, respectively).

Repeated measures analyses indicated a significant improvement in mean scores across modules (*p* < 0.001; Table 1). There was not a significant change between modules 1 and 2 (*p* = 0.73) but there was a significant difference between module 3 and both modules 1 and 2 (**both *p* < 0.001**). Year of training, time elapsed since the last ICU rotation, and number of previous ICU rotations were not significantly associated with improvement in scores from baseline (*p* = 0.70, *p* = 0.21, and *p* = 0.98, respectively). When the analysis was repeated including only residents that completed all modules, the results were similar. Among residents to complete all three modules, average diagnostic accuracy improved from 44.4% to 80.6% (Table 2). Resident’s selection of an appropriate antibiotic regimen was poorest for Module 2, which featured a case of severe community acquired pneumonia.

**Table 1.** Mean scores for users that completed all three teaching modules (*n* = 18).

Module scores	Mean	Standard deviation	Minimum	Maximum	*p*-value
Module 1	57.4%	18.2%	33.3%	93.3%	
Module 2	59.6%	11.7%	38.9%	83.3%	
Module 3	79.9%	12.7%	53.8%	100%	
Difference in score					
Module 2 – Module 1	2.2%	17.9%	−27.8%	27.8%	0.73
Module 3 – Module 1	22.5%	16.0%	−3.1%	53.3%	<0.001
Module 3 – Module 2	20.3%	14.6%	−7.3%	45.7%	<0.001

**Table 2.** Accuracy of correct orders placed by users to complete all three modules (*n* = 18).

Variable	Module 1	Module 2	Module 3
Diagnosis	44.4%	61.1%	80.6%
Antibiotics	94.4%	5.6%	57.9%
Lactate	66.7%	84.2%	94.7%
Vasopressors or IV fluids	61.1%	73.7%	100.0%
Blood culture	94.4%	94.7%	100.0%

Surveys were provided to all participants immediately before and after the session to determine comfort level in caring for critically ill patients and to determine residents’ perception of the modules. Twenty-seven (69.2%) residents completed both surveys. Twenty-one (77.8%) residents reported being moderately or very comfortable caring for critically ill patients before the session compared to 23 (85.2%) after the session (McNemar’s *p* = 0.41). Twenty-five (92.6%) reported the modules as being realistic. When asked which aspects of the training modules were helpful in improving performance, the two most common responses were repetition of modules (66.7%) and immediate feedback provided (48.1%). When asked when the modules would be most helpful, the two most common responses were before an ICU month (85.2%) and during intern year (74.1%). Finally, 85.2% of residents reported the modules were an effective form of teaching with 55.6% reporting they were very effective, and 88.9% of residents reported they would use the technology autonomously if more modules were provided.

## Discussion

In this pilot project, we created a novel educational tool that incorporated real patient data in a teaching electronic medical record format with case-based teaching modules. Learners’ scores improved as they completed successive modules based on iterative feedback built into the cases. We identified gradual improvement in sepsis management: notably diagnosis, lactate measurement and IV fluid or vasopressor delivery (Table 2). We postulated that the improvement seen in module 3 was related to repetition of the cases, which emphasizes that repeated attempts reinforced learning. In Module 2, we found only one resident (5.6%) correctly identified the appropriate antibiotic regimen for community acquired pneumonia. This illustrates an important value of the tEMR in identifying educational priorities within a residency training program with potential to improve the quality and safety of patient care.

Following duty hour regulations, new educational modalities are becoming necessary as trainees are allowed less time in the hospital. CAI is a popular modality utilized by residency programs, yet these tools are standardized and often poorly represent real patient care experiences [1,15]. Virtual patients provide a more realistic opportunity but can be cost and time intensive to develop. Our tEMR combines the positives aspects of both CAI and VPs allowing for potential implementation across a wide spectrum of residency programs while still maintaining a realistic experience. Much of the educators’ labor would be dedicated to identifying ideal teaching cases from the electronic medical record system and developing any supplemental case vignette materials to complement real patient data to optimize learning goals. Importantly, given that residents can be harsh critics of teaching sessions unrelated to direct patient care, our learners overwhelmingly reported that the modules were realistic, an effective form of teaching, and they would use the technology autonomously in the future.

There are limitations to this pilot project that should be identified. Among residents that attended the session, 49% were able to complete all three modules. The reason for lower than expected completion was both technical and time limited. Server issues led to delays at the beginning of the session, which was allotted for two hours. We were not able to reconvene all learners for a subsequent session to complete the modules. We found no significant differences in self-reported comfort levels among users that completed all three modules. This was likely due to the format of the teaching session, as all modules were completed in a two-hour session. We provided links to pertinent medical literature at the end of each module that learners could access on their own time. In this short trial, we did not find significant use of these links (17.9% of resident reported accessing the links) but this was likely due to study design.

Our pilot using the tEMR focused on one disease process, severe sepsis, but one can imagine much broader uses of this education tool. Modules can be created around any disease state and a variety of inpatient and outpatient encounters. Trainee’s performance can be logged and tracked over time documenting readiness for the next stage of supervised or unsupervised patient care. Evaluating if users are accessing the hyperlinks to literature can be used to demonstrate and document behavioral patterns of self-directed learning. Our tEMR has the capability to track where learners move through the chart as well as the order of processing a patient encounter through the EMR. This capability was not assessed in this study. A follow up study could potentially investigate the sequence of events learners move through an EMR while encountering an inpatient admission. The technology does not have to be limited to just graduate medical education. Modules could be used to study ordering practices for other groups of practitioners including fourth year medical students, practicing internists, resident physicians in other specialties, physician extenders, and interprofessional education.

## Conclusion

In summary, we created an educational tool that incorporates real patient data in a mirrored teaching electronic medical record. Our results identify a strategic way to improve resident education in the face of duty hour restrictions. The modules created were well received by internal medicine residents and improved their ordering practices for the care of critically ill patients. The next step in development would be wider availability to trainees with less restraint on time for completion and allowing trainees to complete the modules outside of the classroom on their own schedule. Additionally, repeat testing with similar modules at a future time could be performed to determine if learners retained the knowledge learned in this initial study period.We believe this has great potential given its generalizability and accessibility.

## Supplementary Material

Supplemental_data.zip

## Appendix 1: Gold standard core orders required for Modules.

**Table:**

Module 1: Cholecystitis Total points: 15	Module 2: Severe Community Acquired Pneumonia Total points: 18	Module 3: Obstructing Nephrolithiasis Total points: 13
Diagnosis Septic Shock Cholecystitis Orders Antibiotics: Ampicillin-Sulbactam OR Piperacillin-Tazobactam OR ceftriaxone and metronidazole Triple lumen venous catheter Lactate Urinalysis with culture Blood culture Urine Pregnancy Test CMP OR Hepatic Function Panel CBC Norepinephrine Sequential Compression Devices NPO Ranitidine or Esomeprazole General Surgery Consult	Diagnosis Respiratory failure Severe Sepsis Pneumonia Orders Antibiotics: Ceftriaxone and Azithromycin OR ceftriaxone and Levofloxacin OR ceftriaxone and Moxifloxacin Tracheal intubation Ventilator settings (must have all for credit) Ventilator Mode Ventilator Tidal Volume Ventilator PEEP Ventilator Rate Ventilator FiO2 ABG OR VBG Lactate CBC BMP Sputum Culture Blood Culture Legionella urine antigen Strep pneumoniae urine antigen Ranitidine OR Esomeprazole Enoxaparin OR Heparin Sequential Compression Devices 1-liter fluid bolus	Diagnosis Septic Shock Pyelonephritis Orders Antibiotics: Ampicillin-Sulbactam OR Piperacillin-Tazobactam OR Meropenem Triple lumen venous catheter placement Lactate Urinalysis + Urine culture Blood culture BMP CBC Norepinephrine Sequential Compression Devices Ranitidine or Pantoprazole Urology Consult or Radiology Consult
