# Supplementary figures and images for: A pilot study: a teaching electronic medical record for educating and assessing residents in the care of patients
Source: Med Educ Online. 2018 Mar 6;23(1):1447211. doi: 10.1080/10872981.2018.1447211 (PMC5844037; doi:10.1080/10872981.2018.1447211)

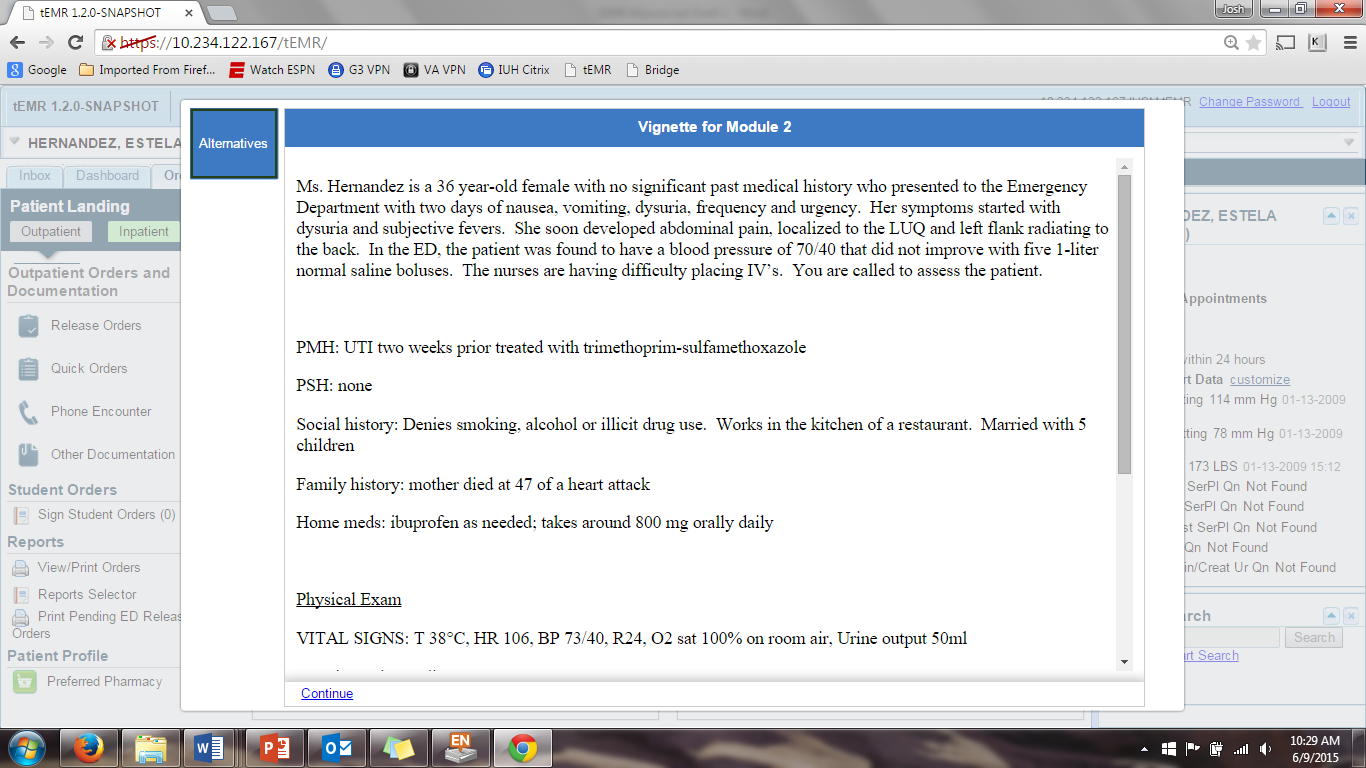

Supplement: Supplemental_data.zip [file ZMEO_A_1447211_SM4839.zip › Supplemental data/33339-209305-1-SP.png]

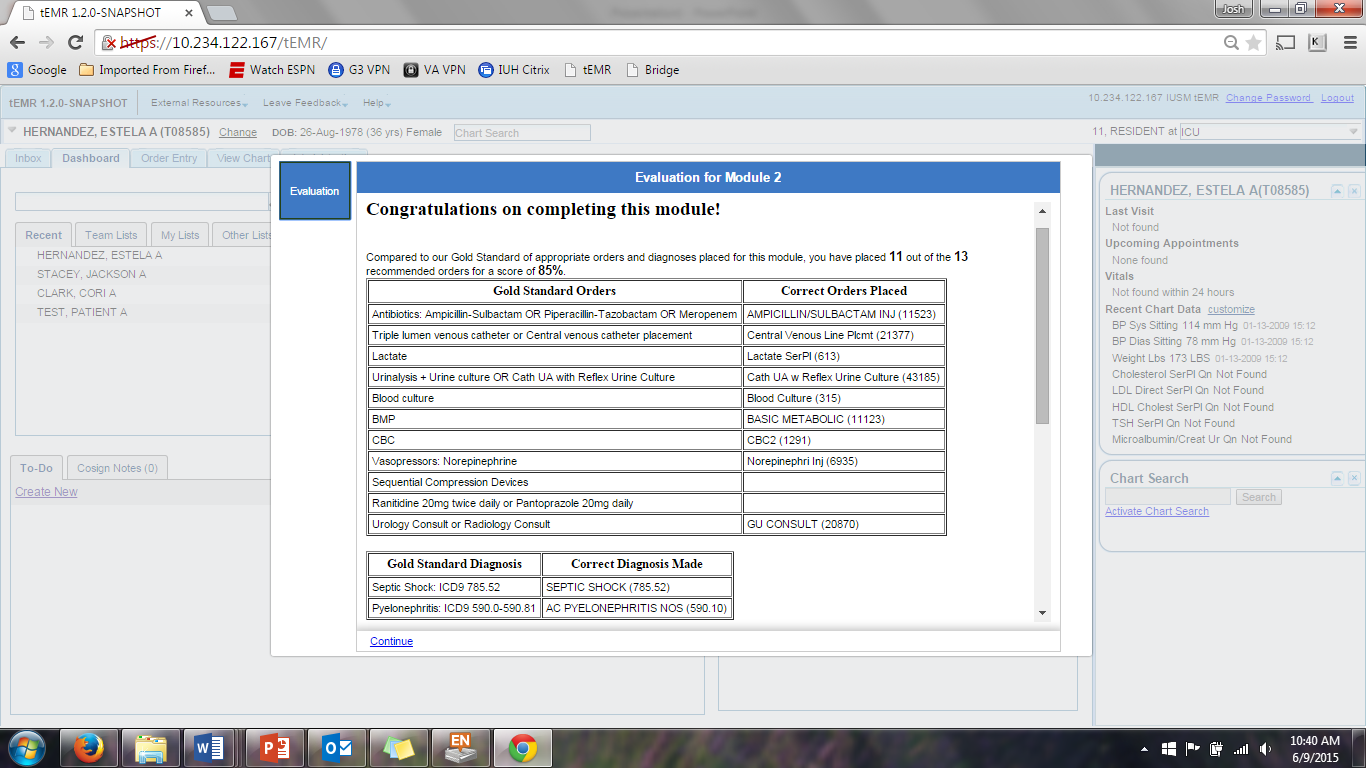

Supplement: Supplemental_data.zip [file ZMEO_A_1447211_SM4839.zip › Supplemental data/33339-209306-1-SP.png]
